# Stage-specific prognostic biomarkers in melanoma

**DOI:** 10.18632/oncotarget.2907

**Published:** 2015-01-10

**Authors:** Yabin Cheng, Jing Lu, Guangdi Chen, Gholamreza Safaee Ardekani, Anand Rotte, Magdalena Martinka, Xuezhu Xu, Kevin J. McElwee, Guohong Zhang, Youwen Zhou

**Affiliations:** ^1^ Department of Dermatology and Skin Science, Vancouver Coastal Health Research Institute, University of British Columbia, Vancouver, British Columbia, Canada; ^2^ Department of Pathophysiology, Basic Medical College, Zhengzhou University, Zhengzhou, Henan, China; ^3^ Bioelectromagnetics Laboratory, Zhejiang University School of Medicine, Hangzhou, Zhejiang, China; ^4^ Department of Pathology, University of British Columbia, Vancouver, British Columbia, Canada; ^5^ Department of Dermatology, Second Affiliated Hospital, Dalian Medical University, Dalian, China; ^6^ Department of Pathology, Shantou University Medical College, Shantou, Guangdong, China

**Keywords:** prognostic biomarker, stage-specific, melanoma, BRAF, MMP2

## Abstract

The melanoma staging system proposed by the American Joint Committee on Cancer (AJCC) (which classifies melanoma patients into four clinical stages) is currently the most widely used tool for melanoma prognostication, and clinical management decision making by clinicians. However, multiple studies have shown that melanomas within specific AJCC Stages can exhibit varying progression and clinical outcomes. Thus, additional information, such as that provided by biomarkers is needed to assist in identifying the patients at risk of disease progression.

Having previously found six independent prognostic biomarkers in melanoma, including BRAF, MMP2, p27, Dicer, Fbw7 and Tip60, our group has gone on to investigate if these markers are useful in risk stratification of melanoma patients in individual AJCC stages. First, we performed Kaplan-Meier survival and Cox proportional multivariate analyses comparing prognostication power of these markers in 254 melanoma patients for whom the expression levels were known, identifying the best performing markers as candidates for stage-specific melanoma markers. We then verified the results by incorporating an additional independent cohort (87 patients) and in a combined cohort (341 patients).

Our data indicate that BRAF and MMP2 are optimal prognostic biomarkers for AJCC Stages I and II, respectively (*P* = 0.010, 0.000, Log-rank test); whereas p27 emerged as a good marker for AJCC Stages III/IV (0.018, 0.046, respectively, log-rank test). Thus, our study has identified stage-specific biomarkers in melanoma, a finding which may assist clinicians in designing improved personalized therapeutic modalities.

## INTRODUCTION

Melanoma is one of the most notorious human cancers with high mortality, due to its aggressiveness and resistance to traditional chemo- and radio-therapies. This disease arises from abnormal melanocyte proliferation, and can occur in any anatomic location containing melanocytes [1]. When diagnosed early, melanoma can be cured by surgical removal. However, late stages of melanoma are often fatal as currently available or developing treatment strategies are only able to prolong life for a few months, expensive and may produce severe adverse effects [2]. The American Joint Committee on Cancer (AJCC) melanoma staging system is currently widely used for melanoma classification, prognostic prediction, and individualized therapy design. This system classifies individual patients into four stages: Stage I and Stage II define primary invasive melanomas, whereas Stage III and Stage IV define local regional and distant metastases, respectively [3].

Several pathological parameters (such as thickness, ulceration and mitotic rate) and serum biomarkers (such as LDH levels) have been taken into account to stratify patients into the AJCC Stages. However, the AJCC system is imprecise when it is applied to assign risks of mortality to individual patients within the specific AJCC stages. It is long recognized that early-stage melanomas are clinically heterogeneous with a subset exhibiting high-risk behaviours. For example, approximate 5% of Stage I melanomas metastasize early and eventually cause death [3, 4]. At present these patients are under-treated since they are placed in clinical surveillance only after surgical excision. Conversely, many patients with stage II melanomas have wide excisions, with a margin of 2 cm or larger, often with high morbidity associated with aggressive surgery. [5]. In contrast, for stage III and stage IV patients, over-treatment with toxic chemotherapies (e.g. Dacarbazine) and biological therapies (interferons) are common [5, 6]. However, the clinical benefit for treating the subset of Stage III/IV patients with these agents is not obvious, and some patients belonging to these advanced stages do survive long periods after diagnosis. With tools such as biomarkers that can further provide prognostic information of patients in each specific AJCC stage, additional degrees of therapeutic individualization can take place, thus potentially significantly improving patients' outcomes. Therefore, AJCC-stage specific biomarkers are urgently needed.

To assist clinicians in further stratifying melanoma patients and estimating probability of disease progression and survival, biomarkers that are able to provide additional information have been extensively evaluated and studied. In the past decades, a significant number of tissue biomarkers have been identified by immunohistochemistry (IHC). But unfortunately, none have been, or are close to being, translated into clinical practice [7–10]. Some of these biomarkers have demonstrated statistical significance as a prognostic marker in a research setting; however, at present, there has been no systematic analysis of molecular biomarkers to identify those capable of refining sub-groupings for individual AJCC stages. Moreover, these tissue biomarkers have not been compared with each other and therefore, it is currently unknown which marker is best for use in a clinical setting.

In this study, we attempted to identify histological markers that are prognostically significant for each individual AJCC Stage. Six previously reported independent melanoma biomarkers, including BRAF, MMP2. P27, Dicer, Fbw7 and Tip60 [11–16] (Table 1), were chosen for this purpose. Using the same set of melanoma tissue microarrays, we found that BRAF expression and MMP2 expression are the best markers for AJCC Stages I and II, respectively, whereas p27 cytoplasm expression is a superior prognostic marker for patients in both Stages III and IV.

**Table 1:** List of 6 markers selected for comparative analysis of prognostic significance for each stage of melanoma

Marker	Full name	Function
BRAF	V-raf murine sarcoma viral oncogene homolog B	Oncogene
MMP2	Matrix metallopeptidase 2	Oncogene
P27	Cyclin-Dependent Kinase Inhibitor 1B	Oncogene
Dicer	Dicer 1, ribonuclease type III	Tumor suppressor
Fbw7	F-box and WD repeat domain containing 7	Tumor suppressor
Tip60	Histone acetyltransferase KAT5	Tumor suppressor

## RESULTS

### Study populations used for biomarker discovery and marker confirmation studies

The clinical databases for six biomarkers previously reported by our group were combined, and the cases with staining scores for all six biomarkers were extracted to form a discovery cohort, permitting the inter-biomarker comparison. This biomarker discovery cohort consisted of 254 patients, including 148 primary melanomas and 106 metastatic melanomas. After identifying stage-specific biomarkers, the samples with expression scores available for individual biomarkers (87 patients, 48 primary melanomas and 39 metastatic melanomas) were studied as an independent cohort. For the validation purposes, the independent cohort was combined with the discovery cohort to form a combined confirmation cohort. In total, 341 patients were used in our study. The distribution and selected demographic characteristics of melanoma patients are listed in Table 2. As shown in Supplementary Figure S1, the 5 year melanoma-specific survival (survival time to melanoma related death, a 5 year time period is used for all the survival time in this study) proportions for Stages I, II, III, and IV are 89.0%, 61.0%, 40.6% and 8.2%, respectively, in a total of 341 patients (Supplementary Figure S1b). The prognosis of each of the AJCC Stages in both the discovery cohort (Supplementary Figure S1a) and in the combined confirmation cohort (Supplementary Figure S1b) is similar and consistent with that reported in studies with larger samples from other laboratories, suggesting that our study patient populations are representative [3, 4].

**Table 2:** Clinicopathologic characteristics of study population of melanoma patients

Variable	Discovery population (*N* = 254)	Validation population (*N* = 341)
Age – yr		
Median	61	60
Range	7–95	7–95
Sex – no. (%)		
Male	148 (58)	204 (60)
Female	106 (42)	137 (40)
Breslow thickness of primary tumor – no. (%)		
≤ 2 mm	61 (41)	80 (41)
> 2 mm	86 (58)	114 (58)
Unspecified	1 (1)	1 (1)
Ulceration of primary tumor – no. (%)		
Positive	48 (32)	58 (17)
Negative	100 (68)	283 (83)
Subtype of primary tumor – no. (%)		
Superficial spreading melanoma	52 (35)	66 (19)
Lentigo maligna melanoma	15 (10)	22 (6)
Acrolentigous melanoma	6 (4)	8 (3)
Nodular melanoma	35 (24)	43 (13)
Others	40 (27)	202 (59)
AJCC stage		
I	55 (22)	73 (21)
II	93 (37)	123 (36)
III	69 (27)	96 (28)
IV	37 (14)	49 (15)

### Expression of six chosen biomarkers is significantly changed during melanoma progression

**Figure 1:**
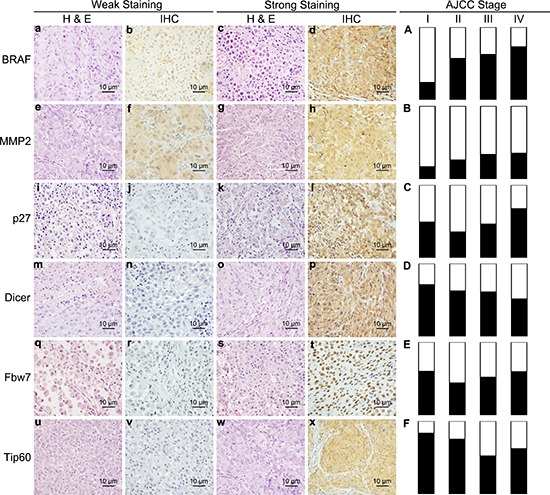
Expression levels of 6 biomarkers are changed across melanoma AJCC Stages **(a–x)** Representative weak and strong immunohistochesistry stain and related haematoxylin and eosin stain images for BRAF **(a–d)**, MMP2 **(e–h)**, p27 **(i–l)**, Dicer **(m–q)**, Fbw7 **(r–u)** and Tip60 **(v–x)**; **(A–E)** Percentage of weak and strong staining in AJCC Stage I, II, III and IV of 6 biomarkers. a-x, bar = 10 μm.

Based on the established tissue microarray (TMA) with 707 biopsies from patients with different stages of melanocytic lesions, our group had previously identified six prognostic biomarkers (Table 1) for cutaneous melanoma [11–16]. To further understand the role of these biomarkers in melanoma progression and to select the best stage-specific prognostic biomarkers, we performed additional analyses on the expression patterns of these six biomarkers in primary and metastatic melanomas. As displayed in Figure 1, BRAF and MMP2 proteins showed a progressive increase from Stage I to Stage IV (Figure 1A, 1B), whereas Tip60 loss is most pronounced in metastatic melanoma (Stage III and IV) as compared to primary melanomas (Stages I and II) (Figure 1F). In contrast, p27, Dicer, and Fbw7 show similar expression changes across different AJCC Stages (Figure 1C, 1D, 1E). Interestingly, alteration of BRAF protein is found to be most dramatic between AJCC Stages I and II (*P* < 0.001, χ^2^ test), and strong BRAF expression accounts for only 23.6% in Stage I, as compared to 57.0% in Stage II (Figure 1A). One possible explanation is that the activation of the MAPK signalling pathway caused by BRAF increase promotes tumor cell growth and proliferation [17, 18], thereby influencing melanoma progression from AJCC Stage I to Stage II. Expression of the cell cycle inhibitor p27^Kip1^ is also negatively regulated by Ras/Raf cascades [19], and we found p27^Kip1^ to be down-regulated in AJCC Stage II, as compared to Stage I (Figure 1C). This change, however, is not statistically significant (*P* = 0.103, χ^2^ test).

### Identification of optimal biomarker candidates for AJCC stages I to IV

To find the best among the 6 biomarkers tested in this study for a specific AJCC Stage, we analyzed the prognostic correlation of these biomarkers by Kaplan-Meier survival analyses and multivariate Cox-regression analyses [20, 21] adjusting for the important clinical variables, such as age, gender, ulceration, and tumor thickness. A discovery cohort of 254 patients was studied, and a single candidate marker for each specific AJCC Stage was selected based on the lowest *P* value of multivariate analyses.

For Stage I, of the six biomakers, BRAF protein expression emerged as a significant prognostic marker based on Log-rank test (Supplementary Figure S2a) and showed the lowest *P* value compared with other markers in the multivariate Cox-regression analysis (Table 3). For Stage II melanomas, both MMP2 and p27 showed significant prognostic values based on survival and Cox-regression analyses (Table 3, Supplementary Figure S3c and S3d), MMP2, however, appeared to have stronger *P* values in both analyses (*P* = 0.004 vs 0.028, survival analysis, Log-rank test; *P* = 0.001 vs 0.004, Cox regression analysis), as compared to p27. For Stage III and Stage IV patients, p27 emerged as having the strongest prognostic significance based on both analyses (*P* = 0.013 and 0.100, Log-rank test, in Stage III and IV, respectively; *P* = 0.024 and 0.068, Cox regression analysis, in Stage III and IV, respectively) (Table 3, Supplementary Figures S4e, and S5e). Moreover, p27 cytoplasm expression was significantly increased in AJCC Stage IV, as compared to Stage III (*P* = 0.037, χ^2^ test) (Figure 1C), suggesting that p27 is an important prognostic factor in advanced melanoma. This data is consistent with our previous report that cytoplasm p27 was significantly associated with melanoma progression and a poorer 5-year patient survival [15].

**Table 3:** Comparison of prognostic value of candidate markers in each AJCC stage of melanoma in discovery patient cohort#

Factor	AJCC I (*N* = 55)			AJCC II (*N* = 93)			AJCC III (*N* = 69)			AJCC IV (*N* = 37)		
	HR	95% CI	*P* value	HR	95% CI	*P* value	HR	95% CI	*P* value	HR	95% CI	*P* value
Age	1.01	0.96–1.06	0.784	1.01	0.99–1.03	0.406	0.99	0.97–1.02	0.504	1.02	0.99–1.06	0.225
Gender	1.19	0.23–6.26	0.834	1.04	0.50–2.20	0.915	1.81	0.93–3.53	0.080	3.05	1.11–8.39	0.031
Thickness	1.79	0.36–8.94	0.479	1.07	1.01–1.13	0.018	-	-	-	-	-	-
Ulceration	-	-	-	3.05	1.45–6.44	0.003	-	-	-	-	-	-
BRAF	3.86	0.81–18.30	0.089*	0.81	0.38–1.70	0.575	0.87	0.46–1.65	0.673	0.91	0.38–2.17	0.824
Dicer	0.43	0.08–2.24	0.312	1.85	0.82–4.18	0.139	0.93	0.48–1.82	0.833	1.59	0.52–4.89	0.421
Fbw7	3.03	0.50–18.25	0.227	1.81	0.89–3.71	0.103	0.68	0.35–1.33	0.263	0.91	0.37–2.23	0.828
MMP2	1.55	0.14–17.75	0.726	3.85	1.69–8.77	0.001*	0.94	0.42–2.08	0.874	0.95	0.36–2.53	0.923
P27	0.41	0.08–2.02	0.274	2.83	1.39–5.78	0.004	2.12	1.11–4.08	0.024*	2.27	0.94–5.47	0.068*
Tip60	0.55	0.06–4.79	0.588	0.41	0.18–0.93	0.033	0.91	0.44–1.90	0.799	0.36	0.11–1.16	0.085

# this cohort of patients has survival data for each of the six biomarkers

* selected marker for confirmaton analysis in expanded patient cohort

### Confirmation of stage-specific melanoma biomarkers

**Figure 2:**
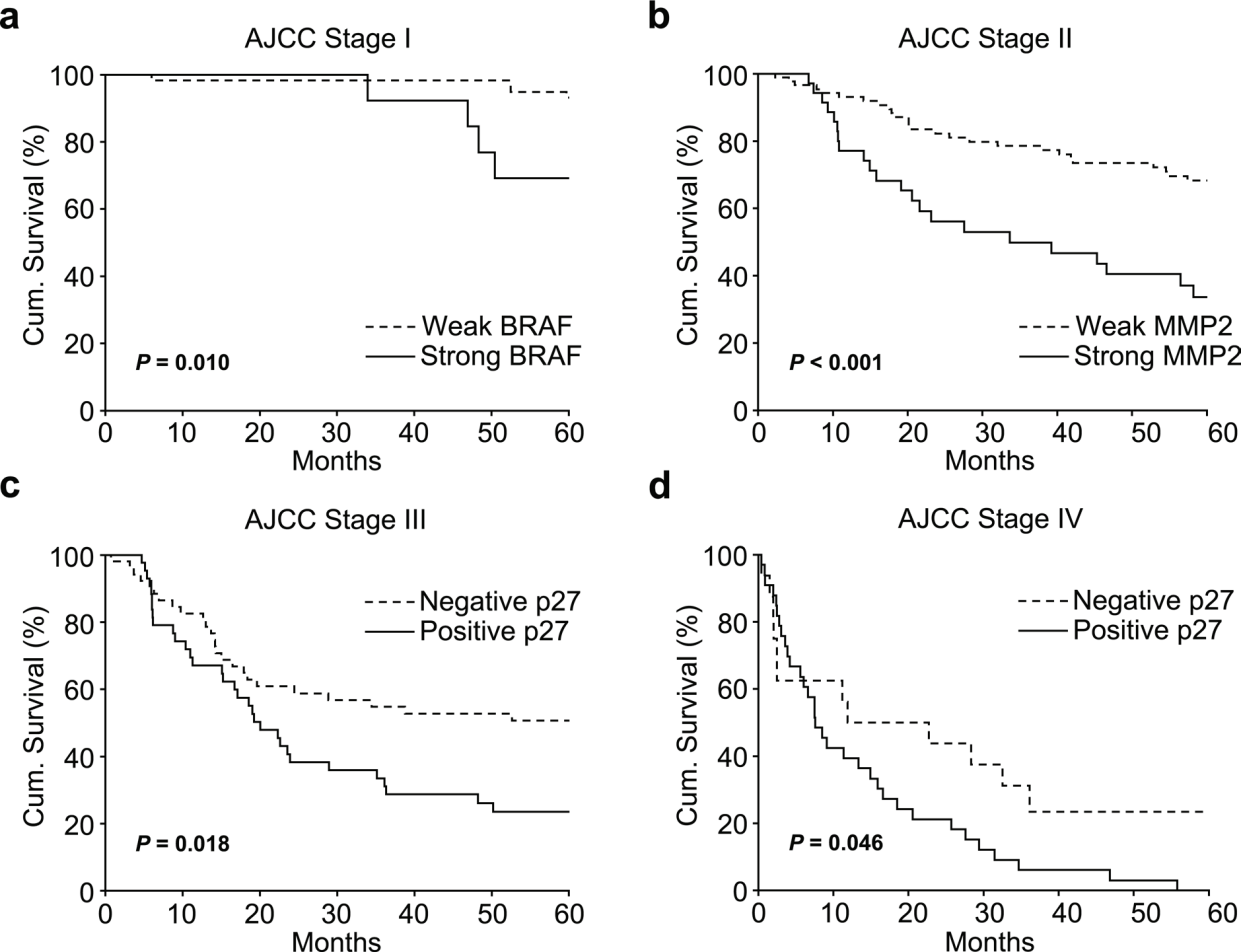
5-Year Kaplan-Meier survival analyses for emerged stage-specific biomarkers in expanded population of melanoma patients **(a)** Strong BRAF expression significantly correlates with worse 5-year survival in AJCC Stage I patients (73 patients, *P* = 0.010, Log-rank test). **(b)** Strong MMP2 expression significantly correlates with worse 5-year survival in AJCC Stage II patients (123 patients, *P* < 0.001, Log-rank test). **(c)** Strong cytoplasm p27 expression significantly correlates with worse 5-year survival in AJCC Stage III patients (95 patients, *P* = 0.018, Log-rank test). **(d)** Strong cytoplasm p27 expression significantly correlates with worse 5-year survival in AJCC Stage IV patients (49 patients, *P* = 0.046, Log-rank test).

To confirm the prognostic value of each stage-specific marker, we performed additional analyses in an additional cohort of patients, as well as a combined cohort (discovery plus additional). Because BRAF expression emerged as the strongest biomarker for Stage I melanoma in the discovery phase, we performed both survival and multivariate Cox-regression analyses based on 73 Stage I patients (analysis was not executed in additional patients, because none of the 18 patients died; Table 4). As shown in Table 5, BRAF possesses a hazard ratio of 4.48 and a *P* value of 0.049 (95% CI: 1.01–19.91). Not surprisingly, 93.2% of patients with weak BRAF expression survived, as compared to 69.2% of those with strong BRAF expression who survived, for at least 5 years (*P* = 0.010, Log-rank test) (Figure 2a).

**Table 4:** Validation of prognostic biomarker for each AJCC stage melanoma in additional melanoma patients#

Factor	AJCC I (*N* = 18)*			AJCC II (*N* = 30)			AJCC III (*N* = 27)			AJCC IV (*N* = 12)		
	HR	95% CI	*P* value	HR	95% CI	*P* value	HR	95% CI	*P* value	HR	95% CI	*P* value
Age	0	NA	1	3.54	0.31–38.4	0.30	0.70	0.23–2.16	0.54	1.73	0.34–8.81	0.51
Gender	0	NA	1	0.43	0.08–2.48	0.35	2.59	0.54–12.5	0.24	0.19	0.02–2.04	0.17
Thickness	0	NA	1	12.1	1.10–132	0.04	-	-	-	-	-	-
Ulceration	0	NA	1	3.86	0.69–21.8	0.13	-	-	-	-	-	-
BRAF	0	NA	1	-	-	-	-	-	-	-	-	-
MMP2	-	-	-	23.1	2.02–264	**0.012**	-	-	-	-	-	-
P27	-	-	-	-	-	-	1.74	0.56–5.39	0.34	4.30	0.61–30.3	0.14

# In addition to the discovery cohort, which has data available for all 6 candidate biomarkers; there are additional patients, for whom data is available for specific markers, but not for all 6 markers.

* No patient out of the 18 in stage I died, thus the Cox regression analysis could not be executed.

**Table 5:** Confirmation of prognostic marker for each AJCC stage melanoma in validation population

Factor	AJCC I (*N* = 73)			AJCC II (*N* = 123)			AJCC III (*N* = 95)			AJCC IV (*N* = 49)		
	HR	95% CI	*P* value	HR	95% CI	*P* value	HR	95% CI	*P* value	HR	95% CI	*P* value
Age	1.01	0.96–1.06	0.710	1.00	0.98–1.02	0.749	0.99	0.98–1.01	0.421	1.00	0.98–1.03	0.674
Gender	1.81	0.41–7.94	0.432	0.86	0.47–1.59	0.638	1.81	0.84–2.43	0.183	3.05	0.97–4.50	0.061
Thickness	1.60	0.35–7.21	0.543	1.05	1.00–1.09	0.045	-	-	-	-	-	-
Ulceration	-	-	-	3.33	1.74–6.34	0.000	-	-	-	-	-	-
BRAF	4.48	1.01–19.91	**0.049**	-	-	-	-	-	-	-	-	-
Dicer	-	-	-	-	-	-	-	-	-	-	-	-
Fbw7	-	-	-	-	-	-	-	-	-	-	-	-
MMP2	-	-	-	3.85	1.39–4.45	**0.002**	-	-	-	-	-	-
P27	-	-	-				1.78	1.05–3.02	0.032	2.36	1.14–4.87	**0.020**
Tip60	-	-	-	-	-	-	-	-	-	-	-	-

Among 123 patients with Stage II melanoma, we performed confirmation analyses for MMP2. MMP2 demonstrated a hazard ratio of 23.1 (*P* = 0.012, 95% CI: 2.02–264) and 3.85 (*P* = 0.002, 95% CI: 1.39–4.45) in additional and combined cohorts, respectively (Table 4, 5), and significantly correlated with worse 5-year patient survival (*P* < 0.001, Log-rank test) (Figure 2).

In Stage III and IV melanomas, the only marker emerging from the discovery phase was p27. Using Kaplan-Meier survival analysis, we confirmed the results for both Stage III and Stage IV populations. In additional patients of Stage III and IV (27 and 12 patients in Stage III and IV, respectively), no significant influence was observed (Table 4), which may be due to the relatively small case number. However, for 95 patients with Stage III melanoma, cytoplasm p27 showed a hazard ratio of 1.78 (*P* = 0.032, 95% CI: 1.05–3.02, Table 5), and p27 expression dramatically affected patient survival (*P* = 0.018, Log-rank test) (Figure 2c). For 49 Stage IV patients, the hazard ratio of p27 was 2.36 (*P* = 2.36, 95% CI: 1.14–4.87, Table 5), and p27 significantly correlated with poor patient survival (*P* = 0.046, Log-rank test, Figure 2). These data confirmed the prognostic significance of BRAF and MMP2 for Stage I, and II respectively, and p27 for both Stage III and IV melanoma patients.

## DISCUSSION

Melanoma is a heterogeneous disease with a series of molecular malfunctions, including defects in cell cycle regulation, cell motility, cell differentiation and cell signaling [22]. Therefore, biomarkers that correlate with tumor biological behavior and reflect molecular signatures hold promise to accurately predict the outcome of melanoma patients. Studies focusing on melanoma biomarkers and high-throughput immunohistochemistry analyses have identified numerous biomarkers with prognostic value. However, there are no prognostic markers used in clinical practice, due to the lack of predictive power and intermarker comparisons to evaluate relative significance of individual markers. Here we examined the relative prognostic significance of multiple histological markers compared with each other based on the same population. Furthermore, we evaluated the histological prognostic markers for each of the individual AJCC Stages. The results of our study show that no one individual prognostic marker is ideal for all AJCC Stages. For Stage I, the best marker is BRAF. For Stage II, the best marker is MMP2, and for Stages III and IV, the best marker is p27. It is currently unknown what biological functional properties of these markers underlie the stage-specific prognostic value. It is possible that the biological challenges to the melanoma cells are different during each step of melanoma progression and metastasis. Further studies are needed to elucidate this possibility.

BRAF is the most commonly mutated gene in melanoma (mutations occur in 50–70%), and mutation of this gene (a majority arising at codon 600) has been considered as the key somatic event in melanoma pathogenesis [23–25]. Targeting mutated BRAF, and consequently shutting down the MAPK signaling pathway, has been directly translated into therapeutic management in melanoma. This strategy has led to approval of two small molecule inhibitors of BRAF by the FDA: vemurafenib and dabrafenib [26–28]. However, the protein expression profiles and the precise prognostic value of BRAF in melanoma remain largely unknown. Our group has previously reported that BRAF expression is increased in primary and metastatic melanomas and predicts worse survival in patients with primary melanoma [11]. Here, we have further shown that increased expression of BRAF protein (regardless of the mutation status) in Stage I melanoma is a significant prognostic marker, in that the higher the BRAF expression the more likely the patient will experience a worse outcome. The correlation between BRAF's prognostic value and the activation of downstream molecules in the MAPK signalling pathway is not fully understood. It has been demonstrated that IHC analysis using anti-BRAF antibody was highly sensitive and specific for detection of BRAF V600E mutation in melanoma [29]. Our data suggest that IHC for BRAF could serve as a useful prognostic marker in the early stage of melanoma as well. Recent studies have demonstrated BRAF to be important for tumor growth and maintenance in melanoma models [30, 31], whereas BRAF seems to possess relatively low oncogenic activity as compared to RAS and PI3K [32, 33]. These observations may have implications of the prognostic value of BRAF expression in Stage I melanomas.

As a well-known oncogene in cancer, MMP2 promotes tumor invasion and metastasis by digesting the extracellular matrix surrounding the malignant tissue [34, 35]. Multiple studies have shown that MMP2 is among the strongest prognostic markers for cutaneous melanoma [10, 36]. Based on comparison of multiple markers in the same database, we further found the prognostic value of MMP2 to be specific to thick primary melanomas (Stage II), whereas for Stage I, its value was not apparent. MMP2 has no apparent prognostic value for metastatic melanomas; the mechanism underlying this phenomenon needs further investigation.

p27 encodes an inhibitor protein regulating the cell cycle G_0_-S phase transition and has been shown to be an atypical tumor suppressor when it is localized in the nucleus [37]. However, in many cancers, p27 is mislocalized, and this mislocalization is associated with a poor prognosis [37, 38]. Multiple mechanisms control the loss of nuclear p27 and increase of cytoplasmic p27 [38]. Chen et al. have shown that an increase of cytoplasmic p27 was associated with poor 5-year survival of metastatic melanoma patients [15]. Here we show that when it is expressed in the cytoplasm, p27 is a predictor for worse prognosis for AJCC Stage III and IV melanoma patients. Interestingly, its value for primary melanoma is not apparent. The fact that it is significant for both Stage III and Stage IV melanomas may indicate that these two melanoma stages face similar physiological challenges as reflected by the expression status of p27.

At present, the AJCC Staging system is used to guide the management of melanoma patients, especially when combined with detailed clinicopathologic characteristics. For Stage I and Stage II patients, ulceration status and individual tumor thickness as well as mitotic figures are used to determine if a sentinel-node biopsy (SLND) is needed along with localized tumor excision. If SLND is positive, the prognosis is much worse [3]. Although the 5-year survival of SLND-positive patients is widely variable, from 64% to 91% [39], SLND is the standard procedure for stratifying primary melanoma patients. Our results showed that histological staining with BRAF (Stage I) and MMP2 (Stage 2) are strong prognostic factors. It is possible that histological staining, which is much easier and readily available, can provide similar or even better value as compared to SLND. It remains to be tested if these histological markers can replace the invasive and technically challenging SLND.

For patients at Stage III and Stage IV, there are at present few factors that can be used to guide clinical management. Very recent studies have shown that melanogenesis, the biochemical process to produce melanin by melanocytes, shortens overall and disease-free survival in patients with Stage III and IV melanoma [40]. The active process of melanogenesis induces cytotoxicity to surrounding tissues (but not melanoma cells), causes genotoxicity and inhibits immune responses, thereby promoting tumor progression [41–43]. As a potential marker for advanced melanoma patients, melanogenesis warrants further investigation. Our results indicate that p27 expression may identify unique patient subgroups of Stage III/IV patients who have low risks for mortality. Therefore, these subgroups might be selected for observation without going through invasive or toxic treatments. In contrast, the high risk group based on p27 expression might benefit from a more proactive treatment regimen. This information may also be a guide in selecting appropriate patient populations to undertake further clinical trials evaluating novel therapies.

Taken together, our study identified stage-specific biomarkers for cutaneous melanoma to further stratify patients into different risk subsets, enabling clinicians to treat selectively those patients more likely to develop distant metastatic disease.

## MATERIALS AND METHODS

### Ethics statement

Our study on archival melanoma biopsies was approved by the Clinical Research Ethics Board of the University of British Columbia. The experiments were performed in accordance with the Declaration of Helsinki guidelines.

### Study patients and tissue microarray

The selection of melanoma tissue blocks and construction of tumor tissue microarrays have been described previously [14]. Briefly, we identified formalin-fixed and paraffin-embedded melanoma biopsies from the 1992–2009 archives of the Department of Pathology, Vancouver General Hospital. Tissue microarrays (TMAs) were constructed as previously described [14]. Due to the core loss, 254 cases could be evaluated for all 6 markers in a discover cohort. However, the lost cores of TMAs were different among markers and additional 87 cases were added to each stage and form a combined cohort for the further confirmation analysis of selected markers.

### Immunohistochemistry and staining intensity assessment

The immunohistochemistry staining protocol and antibodies used for BRAF, Dicer, Fbw7, MMP2, P27 and Tip60 were described in previously published papers [11–16]. Briefly, the staining intensity was scored using the following scale: no staining (0), weak (1), moderate (2), and strong (3). The percentage of positive cells was scored into 4 categories: 1 (0–25%), 2 (26–50%), 3 (51–75%), and 4 (76–100%). The staining intensity and percentage of positive cells were evaluated in a blinded manner by three independent observers (including two dermatologists) simultaneously, and a consensus score was reached for each score. Immunoreactive score (IRS) was used to determine the level of staining by multiplying the scores of staining intensity and the percentage of positive cells. Since cytoplasmic expression of Dicer and P27 were correlated with survival in our previous studies, only cytoplasmic scores were used in this present study. For each biomarker, the x-tile software (version 3.6.1) was used to determine the optimized cut-off points, by selecting the maximal χ^2^ values of the log-rank test for survival between two groups [44].

### Statistical analysis

Initially, to identify candidate stage-specific markers, Kaplan-Meier survival analysis and multivariate Cox proportional hazard model stratified on tumor stage were used to assess the contribution of the different markers in the discovery set. Then, the best candidate marker with lowest *P* value was selected. The results were then confirmed in an independent and a combined patient set. Disease-specific survival was estimated by the Kaplan-Meier method, and survival curves were compared by the Log-rank test. Univariate and multivariate Cox proportional hazards regression models were performed to estimate the hazard ratios (HRs) and their 95% confidence intervals (CI) for the confirmation analysis in expanded patient set. All statistical analyses were carried out using the SPSS version 16.0 software (SPSS Inc., Chicago, IL, USA).

## SUPPLEMENTARY FIGURES


