# Supplementary material for: Stage-specific prognostic biomarkers in melanoma
Source: Oncotarget. 2015 Jan 10;6(6):4180–9. doi: 10.18632/oncotarget.2907 (PMC4414181; doi:10.18632/oncotarget.2907)
Supplement: Supplementary file 1 [file oncotarget-06-4180-s001.pdf]

SUPPLEMENTARY FIGURES

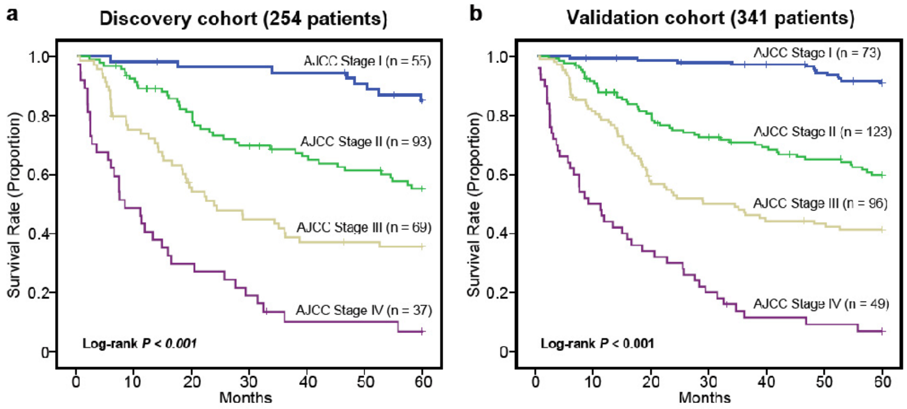

Supplementary Figure S1: 5-year Kaplan-Meier survival analysis of AJCC staging in discovery population (a) and expanded validation population (b) of melanoma patients. (a) Discovery population with 254 patients: proportions of melanoma-specific survival are 85.5% (8 events of death, 47 survive in 55) for stage I, 58.1% (39 events of death, 54 survive in 93) for stage II, 37.7% (43 events of death, 26 survive in 69) for stage III, 8.1% (34 events of death, 3 survive in 37) for stage IV (Log-rank $P < 0.001$); **(b)** Validation population with 341 patients: proportions of survival are 89.0% (8 events of death, 65 survive in 73) for stage I, 61.0% (48 events of death, 75 survive in 123) for stage II, 40.6% (57 events of death, 39 survive in 96) for stage III, 8.2% (45 events of death, 4 survive in 49) for stage IV (Log-rank $P < 0.001$).

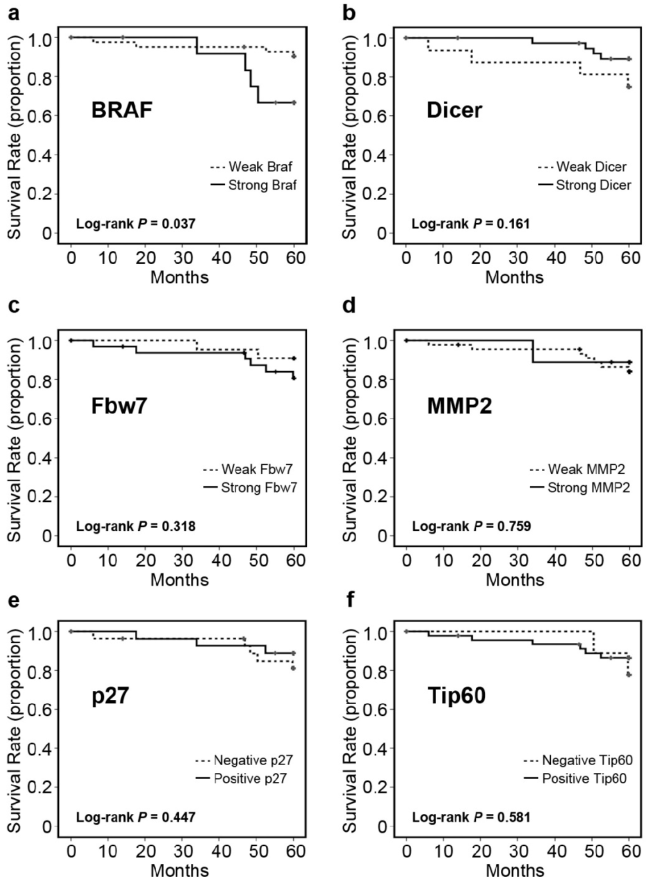

Supplementary Figure S2: 5-year survival analysis of six candidate markers in AJCC stage I melanomas in the discovery set of patients.

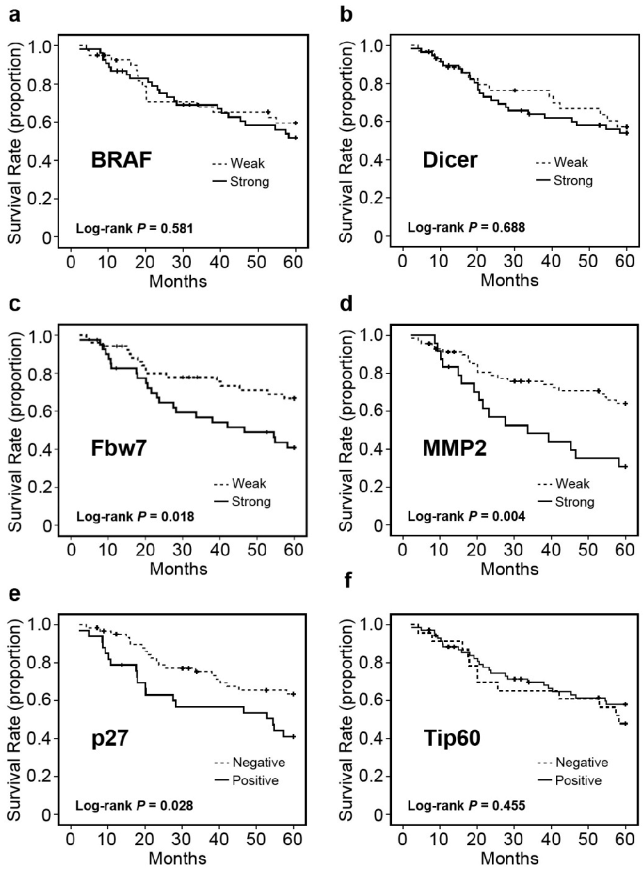

Supplementary Figure S3: 5-year survival analysis of six candidate markers in AJCC stage II melanomas in the discovery set of patients.

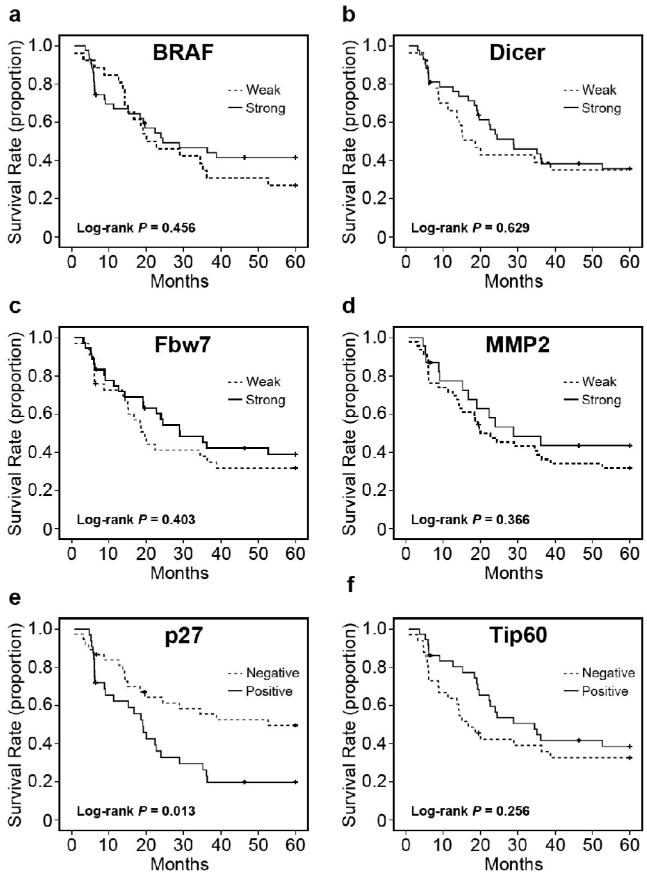

Supplementary Figure S4: 5-year survival analysis of six candidate markers in AJCC stage III melanomas in the discovery set of patients.

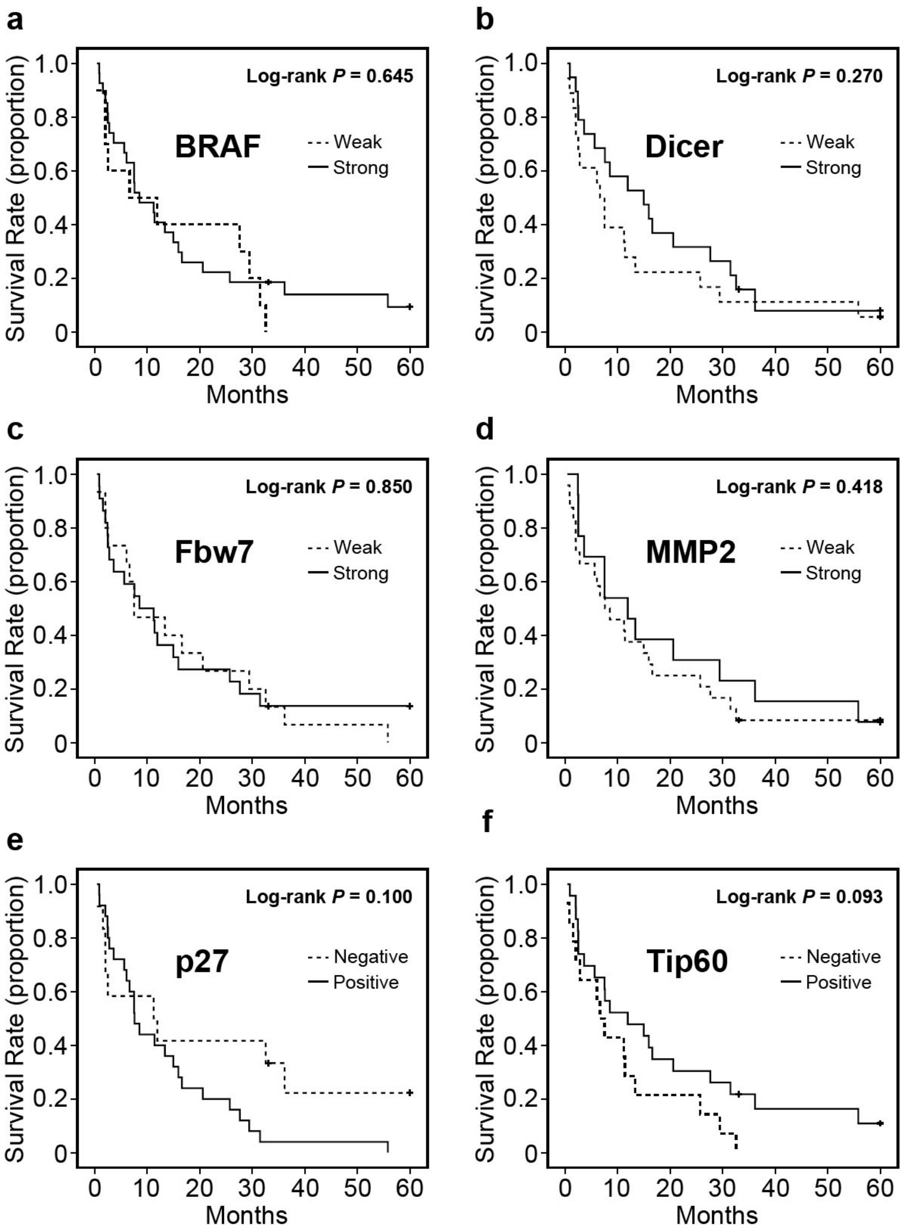

Supplementary Figure S5: 5-year survival analysis of six candidate markers in AJCC stage IV melanomas in the discovery set of patients.
